# mHealth platform improved health worker's compliance to WHO’s IMNCI guideline in Nairobi, Kenya

**DOI:** 10.1093/eurpub/ckac129.423

**Published:** 2022-10-25

**Authors:** M Shah, E Sang, A Afeworki, M McLaughlin, B Finette, L Kwamboka, D Ogaro

**Affiliations:** 1 Global Health, Save the Children US, Washington DC, USA; 2 Health and Nutrition, Save the Children International, Nairobi, Kenya; 3 THINKMD, Vermont, USA; 4 Langata Sub-county Health Management Team, Ministry of Health, Nairobi, Kenya

## Abstract

Poor access to quality health services, especially in urban slums, is a global challenge. Given similar challenges in Nairobi's Kibra informal settlement area, we collaborated with the Langata/Kibra sub-county health management team to conduct a pilot program for improving the quality of child health services delivered by health care providers (HCPs). The pilot introduced a digital mHealth platform to HCPs working in Kibra informal settlement area in Nairobi. This mHealth platform was compliant to WHO's recommended guideline for integrated management of newborn and child illnesses (IMNCI) and was designed to help sick child assessment, diagnosis and management by HCPs. We aimed to determine if using this digital platform, coupled with supportive supervision and community outreach, would lead to improve compliance to the IMNCI guideline for assessment, diagnosis and treatment of sick children. We conducted baseline (February 2019) assessment, trained selected HCPs on the mHealth platform on handheld android tablets, conducted end line (March 2020) and measured any change in HCP's compliance to IMNCI guidelines. Total 89 HCPs were the mHealth platform users during end line assessment. When asked about the choice of antibiotic for treating childhood pneumonia, we found proportion of HCPs who preferred Amoxycillin dispersible tablet, the recommended treatment for childhood pneumonia, increased from 3% at baseline to 38% at end line. Proportion of HCPs who were aware that antibiotics should NOT be used for the management of simple diarrhea increased from 14% (at baseline) to 50% (at end line). At end line, more than 90% HCPs were found compliant in their practice to IMNCI guidelines for sick child assessment, diagnosis and management. These results demonstrate the use of the IMNCI compliant mHealth platforms as a potential important effective way to improve capacity and compliance among HCPs who are serving communities like Kibra informal settlement in Nairobi, Kenya.

**Key messages:**

• WHO recommended IMNCI compliant mHealth platform enables health care providers to offer quality child health care.

• Using mHealth platform to ensure WHO’s IMNCI guideline implementation by health care providers might have potential impact on saving sick children’s lives from preventable deaths.

